# Familial 1q22 microduplication associated with psychiatric disorders, intellectual disability and late-onset autoimmune inflammatory response

**DOI:** 10.1186/s13039-014-0090-7

**Published:** 2014-12-19

**Authors:** Marco Fichera, Rita Barone, Lucia Grillo, Mariaclara De Grandi, Valerio Fiore, Ignazio Morana, Tiziana Maniscalchi, Mirella Vinci, Silvestra Amata, Angela Spalletta, Giovanni Sorge, Salvatore Santo Signorelli

**Affiliations:** Department of Biomedical and Biotechnological Sciences, Medical Genetics, University of Catania, Catania, Italy; Laboratory of Medical Genetics, I.R.C.C.S. Associazione Oasi Maria Santissima, Troina, Italy; Department of Clinical and Experimental Medicine, Child Neurology and Psychiatry, University of Catania, Catania, Italy; Department of Clinical and Experimental Medicine, University of Catania, Catania, Italy; Internal Medicine Unit, Garibaldi Hospital, Catania, Italy; Department of Clinical and Experimental Medicine, Pediatric Clinic, University of Catania, Catania, Italy; Department of Clinical and Experimental Medicine, Medical Angiology Unit, University of Catania, Catania, Italy

**Keywords:** CBCL dysregulation syndrome, 1q22, *LMNA*, *SEMA4A*, *LAMTOR2*, Intellectual disability, CNV, Duplication, Inflammatory disease

## Abstract

**Background:**

Despite the extensive use of chromosomal microarray technologies in patients with neurodevelopmental disorders has permitted the identification of an increasing number of causative submicroscopic rearrangements throughout the genome, constitutional duplications involving chromosome 1q22 have seldom been described in those patients.

**Results:**

We report on a pedigree with seven affected members showing varying degrees of behavioural and emotional disturbances including general anxiety disorder, mood disorders, and intellectual disability. Two adult female patients also showed late onset autoimmune inflammatory responses characterized by alopecia, skin ulcers secondary to inflammatory vasculitis, interstitial lung disease, and Raynaud’s phenomenon. Array-CGH analysis identified in the affected individuals a 290 Kb microduplication in the chromosome 1q22. The rearrangement involves eleven known genes and is not present in the databases of polymorphic copy number variants.

**Conclusions:**

The rearrangement segregates with the neurological clinical features observed in our patients, suggesting that dosage imbalance of one or more genes in this genomic region may lead to the observed phenotype. The association between the microduplication and the inflammatory disease is much less evident. Additional reported patients carrying similar microduplications are needed to clarify this aspect.

## Background

In recent years, chromosomal microarray technologies (CMA) have allowed the identification of an increasing number of submicroscopic chromosomal deletions and duplications, called copy number variations (CNV), associated with a variety of disorders, including intellectual disability, neuropsychiatric disturbances, and congenital developmental anomalies. Indeed, in subjects where no clear genotype/phenotype correlation exists, CMA actually represents the first-tier genome-wide diagnostic test [1].

Despite the worldwide constant increase in CMA testing both for clinical and for research purposes, has allowed an exponential accumulation of knowledge in this field, the clinical interpretation of the CNVs identified during the diagnostic setting may still represents a challenge as many candidate rearrangements are rare variants never associated with a clinical condition. Moreover, as demonstrated by studies on large cohorts of healthy and affected individuals, an increasing number of CNVs are low-penetrant, risk factors for several diseases. In this context, rare CNVs are often classified as VOUS (variant of unknown significance) whose exact role remains to be defined by further studies.

Here we report on a rare 1q22 microduplication identified in a young boy with intellectual disability and psychiatric disturbances. Further clinical and molecular analysis demonstrated that the rearrangement segregated with the disease in the family and that two adult female patients also presented with clear signs of a systemic inflammatory disease. We also discuss the potential role of the microduplication both in the neurodevelopmental and inflammatory disorders.

## Case presentation

**Figure 1:**
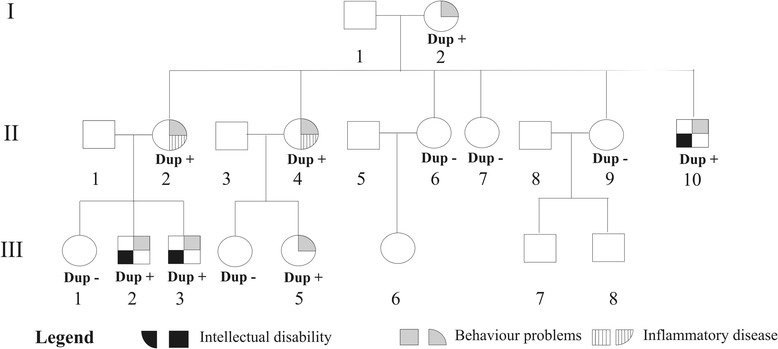
**Pedigree of family members with 1q22 microduplication.** Individuals without genotypes were not available for genetic testing.

**Figure 2:**
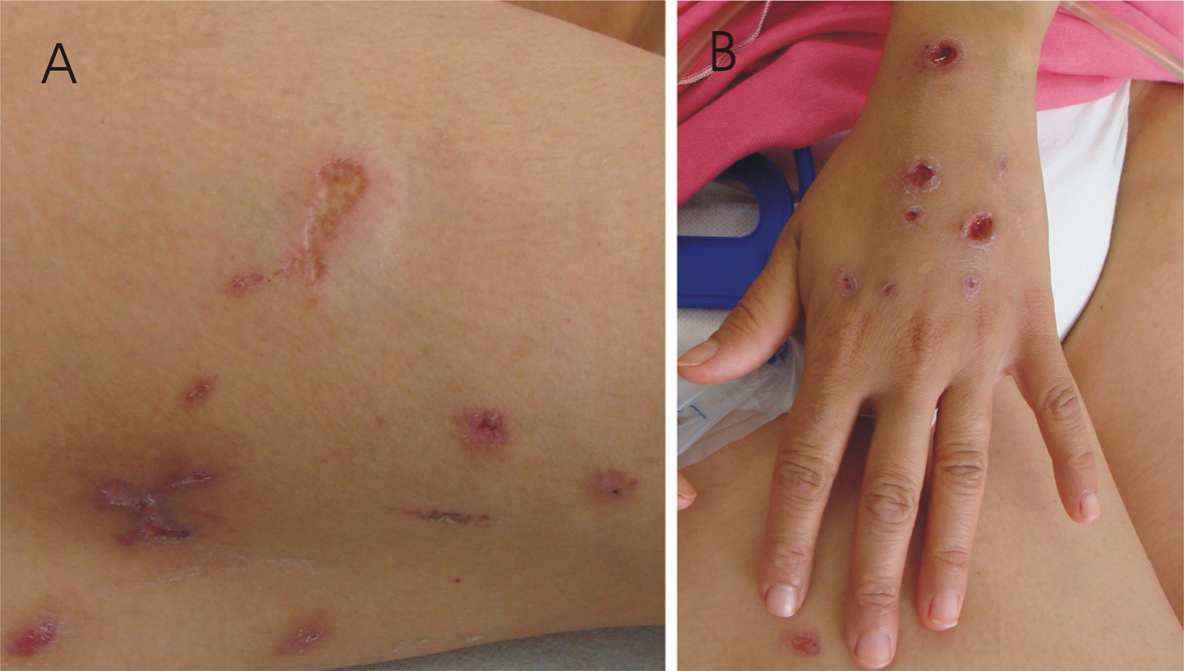
**Skin lesions in patient II-** **2. A)** Lower limbs. **B)** Hands.

Four subjects (3 males), with age ranging from 6 to 16 years were studied. They include one sib pair, a first cousin and one uncle from the maternal side (Figure 1). The proband (III-2), a 11-year-old boy, came to our observation at age 6 because of behavioral disturbances and global developmental delay. He was born to unrelated parents and his pre- and perinatal histories were unremarkable. He walked unsupported at 24 months and produced a two-word sentence after the age 3 years. As a toddler he showed hyperactivity and opposition-defiant disorder (ODD), aggressive and destructive behavior. Physical and neurological examinations were unremarkable. Routine analyses, chromosomal analyses, FMR1 gene molecular analyses and screening for neurometabolic disorders were normal. Brain MRI, EEG, ABR, VEP, EMG and repeated EEG evaluations yielded normal results. He had moderate intellectual disability (WISC-III total IQ score: 46). At present time (age 11 years), he shows a significant impairment of adaptive behavior and psychiatric disturbances with anxiety and depressive symptoms and rule-breaking and aggressive behavior. The younger brother of the proband (III-3) and two other relatives (III-5, II-10) came to our observation because of developmental delay and/or behavioral disturbances. Neurocognitive profile and behavioral features of all studied subjects are reported in Table 1. Cognitive levels were measured by Wechsler Intelligence Scales for Children-III (WISC-III), and/or Leiter-R scales and indicated normal to moderate cognitive impairment. Psychiatric symptoms were featured by Children Behavior Check-list (CBCL) questionnaire filled by both parents and using a semistructured psychiatric interview: K-SADS PL that indicated attention deficit, disthymic disorder, generalized anxiety disorder, oppositive-defiant disorder and conduct disorder with rage episodes. All patients had a CBCL total problem T-score in the clinical range (≥ 70), that was not related to the presence and/or degree of intellectual disability. Internalizing T-scores exceeding the borderline or clinical cut-off points for psychiatric disturbances. In all emotional symptomatology was reflected by abnormal scores in the withdrawn, somatic complain and anxious/depressed scales. In addition, two subjects (III-2, III-5) had significant externalizing symptoms with clinically abnormal scores in the aggressive and rule-breaking behavior symptom scales and they encountered criteria for CBCL-Severe Dysregulation profile defined by a score of ≥210 (2 SDs) on the sum of the Attention, Aggression, and Anxious/Depressed CBCL scales [2]. Adult subjects (I-2, II-2, II-4) refused a formal psychiatric interview although they clearly manifested rapid changes of mood and restless speaking and had been following medical treatment for depressive disorder. Moreover, individual II-2 presented to our clinic with recurrent fever (38°-39°C), continuous pain, several superficial skin damages both in the arms and legs (Figure 2). Skin capillaries biomicroscopy (i.e. capillaroscopy) did not show any pathological findings while a skin biopsy demonstrated clear signs of arteriolar vasculitis. The corticosteroid therapy and vasoactive drugs (Pentoxyphilline) were effective both in reducing the pain and also in recovering skin lesions. Her sister (II-4) suffered from a chronic inflammatory disease with microcirculatory damage (i.e. progressive systemic sclerosis) and treated with anti-inflammatory drug (low dose of cortichosteroids).

**Table 1:** **Neurocognitive and psychiatric symptoms** (**CBCL T**-**scores**)

**Patient/** **age**	**III-** **2/** **11y**	**III-** **3/** **6y**	**III-** **5/** **10y**	**II-** **10/** **16y**
**IQ**	**46**	75	88	**52**
**Total**	**75**	**70**	**78**	**70**
**Internalizing**	**68**	**65**	**75**	**70**
**Externalizing**	**77**	59	**76**	61
**Anxious/** **depressed** **(int)**	64	57	**80**	**68**
**Withdrawn** **(int)**	**68**	**66**	60	**78**
**Somatic complaints** **(int)**	64	64	**73**	61
**Social problems**	**70**	**70**	**75**	**77**
**Thought problems**	**67**	**70**	**83**	63
**Attention problems**	**71**	**69**	**84**	62
**Rule–** **breaking behavior** **(ext)**	**80**	53	**73**	57
**Aggressive behavior** **(ext)**	**75**	61	**78**	63

Borderline cut-off: score: ≥ 65; Clinical cut-off score: ≥ 70.

Int, symptom scale included in the internalizing broad-band score; Ext, symptom scale included in the externalizing broad-band score.

(Abnormal scores are in bold character).

## Results

**Figure 3:**
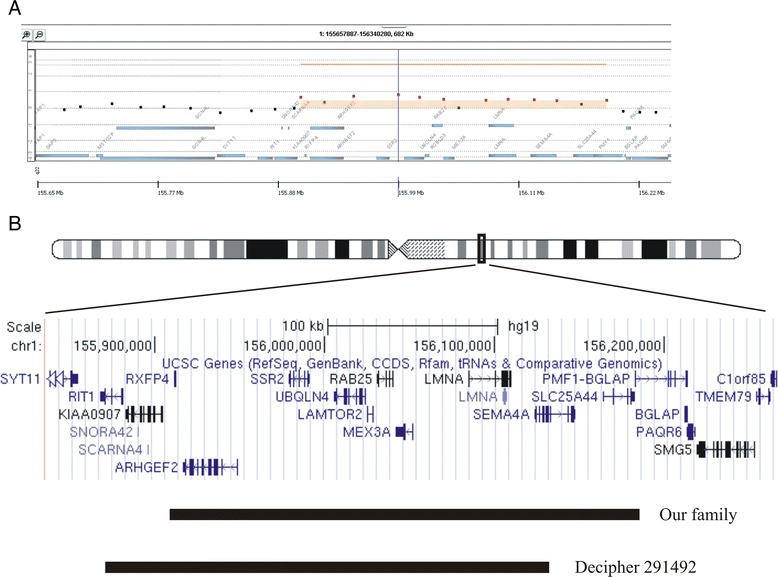
**Array-** **CGH analysis in the proband. A)** Array-CGH profile revealing the 1q22 microduplication. Red dots represent duplicated probes. **B)** Schematic representation of the genomic background of the 1q22 microduplication adapted from the human genome browser (http://genome.ucsc.edu/).

Conventional G-banding karyotype analysis of the propositus (III-2) showed normal male karyotype (46, XY), while microarray analysis revealed an interstitial microduplication of about 290 kb in the chromosome 1q22: arr 1q22 (155.901.586x2, 155.907.817-156.195.054x3, 156.210.558x2) hg19) (Figure 3A). The duplication was also present in Individuals I-2, II-2, II-4, II-10, III-3, and III-5 while it was absent in Subjects II-6, II-7, II-9, III-1, and III-4. The remaining members of the pedigree were not tested. The microduplication, encompassing eleven known genes, was confirmed by home-designed MLPA assay (data not shown). FISH analysis performed in Patient II-2 only showed specific signals on 1q22 chromosome for test probe and on 1p36.33 chromosome for the control probe suggesting a tandem arrangement of the duplication (data not shown) .

## Discussion

Constitutional duplications involving chromosome 1q22 have seldom been described. The familial 1q22 microduplication here reported segregates with the core clinical features of the affected members characterized by intellectual disability and/or psychiatric disturbances including general anxiety disorder, and mood disorders. Interestingly, two adult affected females also showed late onset (first clinical manifestations in their late twenties) autoimmune inflammatory responses characterized by skin ulcers secondary to inflammatory vasculitis, interstitial lung disease, and Raynaud’s phenomenon. These late onset inflammatory traits are neither present in the younger affected family members, nor in the maternal grandmother of the propositus either suggesting that the 1q22 microduplication has an age-related penetrance or it is unlinked to the inflammatory trait. At the time of writing, comparable or larger duplications were not listed in DGV whereas a similar, overlapping microduplication (Figure 3B) was reported in the Decipher database (https://decipher.sanger.ac.uk/index) in an individual with downslanted palpebral fissures, joint hypermobility, microcephaly, and scapular winging. The rearrangement was also present in his, similarly affected, mother. Chan et al. [3] described a constitutional 1q (q11q22) duplication identified by comparative genomic hybridisation in a patient with T lymphoblastic lymphoma/leukaemia with no other developmental defects. Interestingly, the rearrangement was also present in his healthy mother and sister. While these findings suggest that the duplication of this region may be tolerated, it should be noted that the precise boundaries and the exact gene content of the rearrangement were not defined.

The rearrangement identified in our study involves eleven known genes (*ARHGEF2*, *RXFP4*, *LAMTOR2*, *SSR2*, *RAB25*, *UBQLN4*, *MEX3A*, *LMNA*, *SLC25A44*, *PMF1*, *and SEMA4A*). Review of the literature led us to consider *LAMTOR2*, *LMNA*, and *SEMA4A* as the strongest candidate genes, although we cannot exclude that dosage imbalance of other genes implicated in the rearrangement may contribute to the disease.

The p14 protein, encoded by *LAMTOR2*, belongs to the LAMTOR complex which is involved in the activation of the extracellular signaling-regulated kinase (ERK1/2) and the mTOR complex 1 (mTORC1). This protein is also active in the regulation of endosomal trafficking, growth factor signaling and cell proliferation [4]. A homozygous *LAMTOR2* mutation was found in the affected members of a family segregating a primary immunodeficiency syndrome, highlighting the important role of the p14 protein in the immunological processes [5]. The mTOR pathway holds an important immunoregulatory function as it activates conventional T cell and the proliferation of regulatory T cells. Several reports have recently demonstrated that rapamycin, a potent inhibitor of mTOR, may attenuate autoimmune responses reducing lymphoproliferation [6]. However, there are no clear evidence that activation of mTOR may trigger autoimmune manifestations. Recent studies have suggested a link between mTor signaling and synaptic plasticity, memory and neurological disease raising the question whether *LAMTOR2* gene duplication and a subsequent mTOR pathway dysregulation may contribute to the neuropsychiatric traits observed in our patients [7].

Through the use of alternative RNA splicing mechanism the *LMNA* gene codes for A-type lamin proteins, essentially lamin A and C. These two proteins are the main structural elements of the nuclear envelope that protects the nucleus from mechanical stress [8]. Besides this role in the maintenance of the structural integrity of the nucleus, lamin proteins are also important in several biological processes such as DNA replication and chromatin organization [9]. While several missense or truncating mutations of the LMNA gene are associated with muscular diseases, lipodistrophy syndromes and peripheral neuropathy, a specific synonymous mutation (G608G) is the most common mutation identified in the premature aging syndrome Hutchinson-Gilford progeria (HGPS MIM 176670), a severe multisystemic disorder characterized by short stature, low body weight, early loss of hair, lipodystrophy, scleroderma, and cardiovascular defects [10]. The G608G mutation activates a cryptic splice site in exon 11 leading to an abnormal mRNA that encodes a protein called progerin which lacks the ZMPSTE24 cleavage site and remains farnesylated. The main pathophysiological mechanism in HGPS seems related to the toxicity of the farnesylated progerin which cannot incorporate normally in the nuclear lamina causing nuclear morphology defects and changes in gene expression and DNA repair [11]. Homozygous or compound heterozygous mutations in the *ZMPSTE24* gene have been identified in patients affected by HGPS-like disorder such as restrictive dermopathy (RD 275210) and mandibuloacrodysplasia with type B lipodystrophy (MADB; 608612). Either a complete (RD syndrome) or partial (MADB syndrome) loss of function of ZMPSTE24 leads to an accumulation of farnesylated prelamin A which cannot produce mature lamin A. These findings underline how both accumulation of mutant farnesylated progerin and wild-type farnesylated prelamin A are toxic. Interestingly, our patients show clinical signs that also occur in the progeroid-like syndromes such as vascular damage, scleroderma, and alopecia. These observations raise the question of whether gene dosage increase of *LMNA* in our patients may perturb the metabolism of lamin A leading, at least in certain tissues, to an accumulation of the farnesylated prelamin A which in turn produces a tissue damage.

While duplications of lamin B coding gene, *LMNB1* leads to adult-onset autosomal dominant leukodystrophy (ADLD) [12], duplications of the *LMNA* locus has so far not yet been associated with human diseases. However, some reports have recently demonstrated using cellular models that small perturbations in the metabolism of wild-type lamin A induce nuclear defects and result in a progeroid phenotype. Candelario et al. [13] showed that overexpression wild-type lamin A in normal human cells result in nuclear membrane defects and decreased replicative lifespan. These findings have also been confirmed by Huang et al. [14] in fibroblast cell lines overexpressing *LMNA* and showing telomere shortening and nuclear damages. The authors also hypothesized that duplications affecting the LMNA locus may result in progeroid-like phenotypes in carrier individuals. Taken together these observations suggest that the LMNA gene duplication may contribute to the clinical features of our patients.

The *SEMA4A* gene encodes a member of the semaphorin family whose members are involved in axon guidance, morphogenesis, carcinogenesis, and immunomodulation. Mutations in that gene have been associated with retinitis pigmentosa (OMIM 610282) and cone dystrophy (OMIM 610283). *SEMA4A* plays an important role in T cell activation and Th1 differentiation, contributing to adaptive immunity. Although its exact role in autoimmune disease is still to define, studies on experimental autoimmune encephalomyelitis and myocarditis have demonstrated a critical role of *SEMA4A* by regulating T-cell differentiation [15]. Moreover, a recent study suggests that *SEMA4A* is involved in the pathogenesis of Multiple Sclerosis and that high levels of Sema4A are associated with a more severe clinical manifestation of the disease [16].

## Conclusions

The 1q22 microduplication that here we report belongs to a group of candidate CNVs including very rare, often inherited, imbalances which are both not present in the general population and never reported in the literature. Although caution is always necessary in the clinical interpretation of those CNVs, in our family the rearrangement segregates with the neurological clinical features observed in our patients, strengthening its potential pathogenic role. On the other hand, the association between the microduplication and the inflammatory disease is much less evident since the late onset of the disease prevents any genotype-phenotype correlation in the youngest members of the pedigree. Clinical follow-up of these individuals and additional reported patients carrying similar microduplications are needed to clarify this aspect.

## Methods

Array-CGH/SNP analysis was performed using the CytoChip Oligo SNP 4x180K kit (Bluegnome Ltd) containing about 150,000 CGH probes and 27,000SNP probe providing an average resolution of 25 kb. Normal Human male and female DNAs (Agilent Technologies) were used as references. Array images were processed by the Feature Extraction software (Agilent Technologies) and CNVs calls were identified with the Genomic Workbench software 7.0 (both from Agilent Technologies) using the aberration detection statistical algorithm ADM-2 (sensitivity threshold 4). CNVs presenting less than 4 consecutive probes with an absolute value less than 0.4 or present in at least 3 independent studies in the database of Genomic Variants Database of Genomic Variants (DGV: http://dgv.tcag.ca/dgv/app/home), were filtered out from the rest of the analysis. Fluorescent in situ hybridization (FISH) experiment was performed on metaphase chromosome by standard techniques using BAC insert clone RP11-172I6 mapping inside the duplicated region and RP11-54O7 as control clone for the 1p chromosome (BlueGnome, Technogenetics). Multiple-ligation-probe amplification (MLPA) A locus specific assay was designed and performed in the propositus, in the other members of the pedigree and in ten normal individuals (reagents and enzyme were from MRC-Holland, Amsterdam, Netherlands). Amplicons were recognized and quantified by capillary electrophoresis on an ABI 3130 GeneticAnalyser (Applied Biosystems, Foster City, CA). The tracing data were then normalized by dividing each probe’s peak area by the average area of all peaks of the sample and then dividing this value by the average normalized peak’s area of the corresponding locus of all the samples.

## Consent

Written informed consent was obtained from patients or their parents for publication of this paper and any accompanying images.

## Abbreviations

CMA: Chromosomal microarray technologies
CNV: Copy number variant
VOUS: Variant of unknown significance
ODD: Opposition-defiant disorder
MRI: Magnetic resonance imaging
EEG: Electroencephalogram
ABR: Auditory brainstem response
VEP: Visual evoked potential
EMG: Electromyography
CBCL: Children behavior check-list
Kb: Kilobases
CGH: Comparative genomic hybridization
MLPA: Multiple-ligation-probe amplification
FISH: Fluorescent in situ hybridization
DGV: Database of genomic variants.
